# Performance, Determinants, and Acceptability of a Clinical Pharmacy Assessment in Hospital Pharmacy Education

**DOI:** 10.3390/pharmacy14040090

**Published:** 2026-06-24

**Authors:** Sébastien Chanoine, Quentin Perrier, Elisa Vitale, Arnaud Tanty, Benoît Allenet, Pierrick Bedouch

**Affiliations:** 1 University Grenoble Alpes, Pharmacy Department, Grenoble Alpes University Hospital, F-38043 Grenoble, France; qperrier@chu-grenoble.fr (Q.P.); evitale@chu-grenoble.fr (E.V.); atanty@chu-grenoble.fr (A.T.); ballenet@chu-grenoble.fr (B.A.); pbedouch@chu-grenoble.fr (P.B.); 2 University Grenoble Alpes, CNRS, UMR 5525, VetAgro Sup, Grenoble INP, CHU Grenoble Alpes, TIMC, F-38041 Grenoble, France; 3 University Grenoble Alpes, INSERM U1209, CNRS UMR 5309, Institute for Advanced Biosciences (IAB), Team of Environmental Epidemiology Applied to Development and Respiratory Health, F-38700 La Tronche, France

**Keywords:** clinical pharmacy, pharmacy education, formative assessment, experiential learning, competency-based education

## Abstract

*Background:* Pharmacy students in France complete an equivalent six-month full-time hospital placement during the fifth year of their university curriculum. At our school, it includes a clinical pharmacy within a medical ward, with daily supervision by a clinical pharmacist and a pharmacy resident. This training has been strengthened by the introduction of a workplace-based formative assessment conducted at the end of the clinical pharmacy rotation, alongside weekly clinical case discussions at the school, culminating in an end-of-year oral assessment. *Objective*: To assess the performance, determinants, and acceptability of this assessment model. *Methods:* We conducted a retrospective, single-center study over ten academic years (2013–2023). The evaluation combined three complementary components: the workplace-based clinical assessment based on real patient interactions, the case-based oral assessment, and students’ satisfaction. *Results:* Nearly one thousand students were included. Students’ performances remained stable over time. Higher scores were observed among students with prior clinical experience and those enrolled in hospital-focused training pathways. Student satisfaction was high, particularly in settings with direct pharmaceutical supervision, which was strongly associated with improved perceived learning, engagement, and supervision quality. *Conclusions:* Beyond performance measurement, this model appears to foster clinical reasoning, professional development, and student engagement, suggesting its relevance for competency-based pharmacy education.

## 1. Introduction

Pharmacy curriculum in France take place within the 24 schools spread across the country [1]. After successfully completing the First Common Year of Health Studies, the pharmaceutical academic pathway is structured into three cycles. The first two correspond to the General Pharmaceutical Sciences Training Diploma and the Advanced Pharmaceutical Sciences Training Diploma. These lead to the choice of a specialization: community pharmacy, hospital pharmacy, medical biology, research, or industry, culminating in the state diploma of Doctor of Pharmacy [2].

In addition to theoretical, methodological, applied, and practical teaching, students must complete various types of internships to deepen their knowledge and acquire professional skills. In the United States, where active pedagogical research has demonstrated the utility of pharmacy internships for students and healthcare institutions, these professionalizing internships represent a significant part of the curriculum, especially in hospital and community pharmacy settings [3].

In France, three internships are mandatory during the first two cycles, but optional discovery internships are also available [4]. The four-week full-time initiation community pharmacy internship allows students to discover the role of the community pharmacist, learn about legislation and magistral and officinal preparations, and gain initial experience of patient contact. The application internship, conducted in a community pharmacy or hospital ward in the third and fourth years, enables students to apply their learning to real-life situations. The minimum duration is two full weeks. Finally, the hospital internship consists of work equivalent to six months of full-time employment at the hospital during the fifth year of study.

Since the 1980s, the French fifth year of study has been a crucial step and has evolved to meet the requirements of contemporary practice. The aim of this training year at the hospital is to familiarize students with the realities of hospital practice, strengthen their understanding of clinical pharmacy, and prepare them for their future professional responsibilities. However, despite its importance, the effectiveness of the fifth hospital-university year and hospital internships could be improved. Previous studies have raised questions about supervision, the relevance of activities proposed, and the integration of students into hospital wards [5,6,7]. Despite the mandatory nature of hospital internships, several gaps persist in the French curriculum, notably a lack of standardized clinical supervision and limited integration of students into multidisciplinary teams. Traditional evaluations often focus on theoretical knowledge rather than the ability to manage complex, real-patient scenarios. Our model addresses these gaps by shifting from a passive observation role to an active, competency-based formative assessment. Few investigations were conducted among pharmacy students to understand their perceptions and expectations regarding this training year [8,9].

In this study, we proposed evaluating students’ success through an assessment mode involving professional situational scenarios at the hospital, based on their records and their satisfaction with their internship in clinical services during this fifth hospital-university year, according to their chosen specialization.

## 2. Material and Methods

### 2.1. Formation at the School of Pharmacy During the Fifth Year of University Hospital Studies

**Figure 1:**
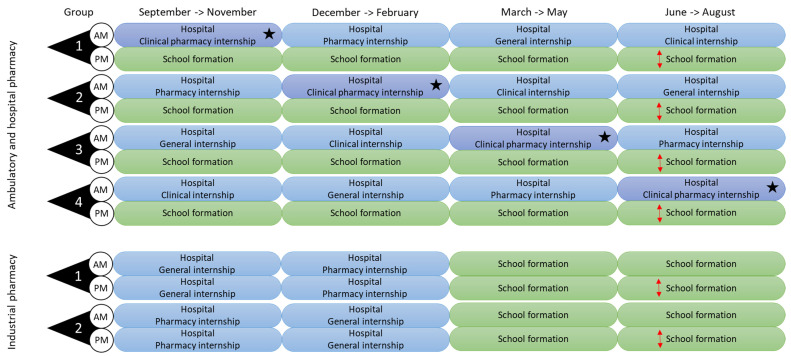
Formation during the fifth year at pharmacy school. AM: ante meridiem; PM: post meridiem; stars indicate the moment of the workplace-based clinical assessment, double arrows indicate the moment of the case-based oral assessment, clinical pharmacy internships with direct pharmacist supervision are indicated in dark blue, other hospital internships in light blue and school formations in green.

In France, the organization of the fifth year of pharmaceutical studies depends on the student’s chosen training pathway (community pharmacy, hospital pharmacy, research, or industry) and can vary from one school to another. At our school, students in the community and hospital tracks complete 3-month part-time (morning only) internships in four different areas, throughout the academic year. Students in the research and industry tracks complete two 3-month full-time internships during the first semester. The remaining time is devoted to the formation related to their field and to a weekly common training session (case-based oral assessment) (Figure 1).

Internships are divided into four categories: senior clinical pharmacy internship, non-senior clinical pharmacy internship, critical care/biology internship, and pharmacy internship. The senior clinical pharmacy internship includes diabetology, infectiology, nephrology, geriatrics, hepato-gastroenterology, hematology, pneumology, rheumatology, pediatrics, hematology day hospital, cardiological follow-up care, and rehabilitation. The non-senior clinical pharmacy internship includes cardiology, gerontology center, digestive surgery, pediatric surgery, vascular surgery, vascular medicine, internal medicine, neurology, medical oncology, thoracic oncology, psychiatry, emergency, post-emergency, and intensive care units. The pharmacy internship includes drugs dispensation, pharmaceutics, medical devices, and sterilization. Each pharmacy and hospital student completes a placement in each category. The order in which the placements are completed is described in Figure 1.

### 2.2. Data

We conducted a single-center, retrospective analysis of hospital internships of 5th-year pharmacy students at our school over a period of ten academic years (2013–2023).

### 2.3. Workplace-Based Clinical Assessment

Students in community pharmacy and hospital fields have, at the end of their senior clinical pharmacy internship, a workplace-based formative assessment. The objective is to assess the knowledge and skills acquired during their internship. Specifically, the evaluation consists of an interview with a patient in order to carry out medication reconciliation upon hospital admission under the supervision of a senior pharmacist and a pharmacy resident. Both independently evaluate the student using a competency assessment grid (graded from 1: insufficient to 5: exceptional), divided into three areas: (i) gathering appropriate information, patient relationship, and communication skills (theme 1), (ii) evaluation of patient therapeutic management (theme 2), and (iii) developing and communicating pharmaceutical opinion (theme 3). The senior pharmacist and pharmacy resident discuss each variation in the competency score until they reach a consensus. Finally, the notation of each theme is pondered to obtain the student’s global evaluation.

### 2.4. Case-Based Oral Assessment

During their fifth year, all students receive weekly training in presenting a clinical case and its pharmaceutical analysis, in accordance with the guidelines provided at the beginning of the year. Each presentation is graded and contributes to the continuous assessment grade. At the end of the academic year, each student takes an oral examination designed to assess his or her knowledge and skills based on a clinical case chosen from those presented during the year. The examination, administered by a jury of two pharmacists, consists of two parts: a 10 min analysis of the clinical case by the student, followed by a 5 min question-and-answer session. The final grade comprises the continuous assessment grade (20%) and the final examination grade (80%).

### 2.5. Students’ Satisfaction

Students are encouraged to evaluate their internship at the end of each one regarding three criteria: the quality of their supervision, the impact and the added value of a pharmacist student in this internship, and the acquired knowledge. Each criterion is scored from 1 (very bad) to 6 (very good).

### 2.6. Statistical Analysis

Categorical variables were expressed as frequencies (and percentages), and quantitative variables as medians (with 25th–75th percentiles). Chi-square and McNemar tests were used to compare categorical variables. Continuous variables were compared with non-parametric tests, i.e., Kruskal–Wallis test followed by Dwass–Steel–Critchlow–Flinger post hoc test for pairwise comparisons, and Mann–Whitney test for independent samples comparisons. A *p*-value < 0.05 was considered significant. Analyses were performed with JAMOVI^®^ software version 2.3.2 (the Jamovi project, 2020).

## 3. Results

### 3.1. Population Characteristics

**Figure 2:**
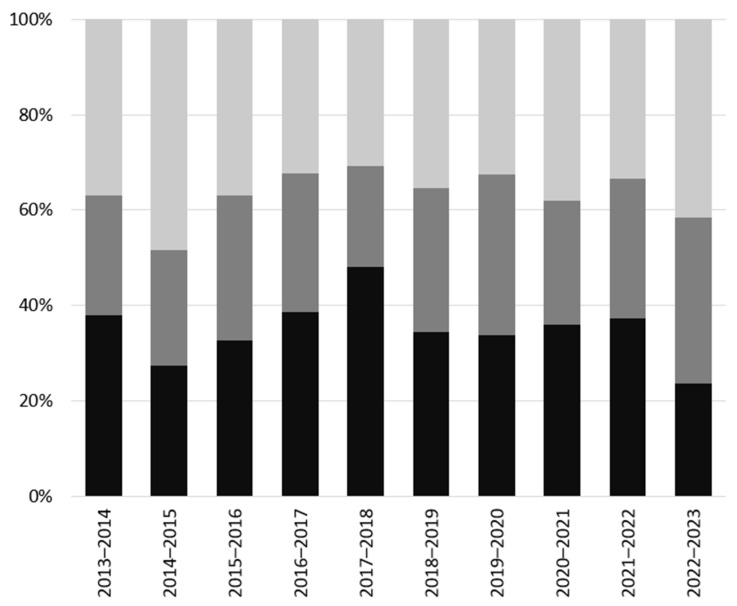
Study track repartition throughout years. Black: hospital pharmacy students, dark gray: industrial pharmacy students, light grey: ambulatory pharmacy students.

From 2013 to 2023, 927 students were trained successively: 231 from the industry track (24.9%), 339 from the hospital track (36.6%), and 357 from the community pharmacy track (38.5%). The distribution of students according to their chosen training pathway remained stable throughout years (*p* = 0.14) (Figure 2). Among them, 917 completed the workplace-based clinical assessment (10 missing data, as some students repeated the year), and 598, from the community and hospital pharmacy tracks, were assessed as part of the case-based oral assessment (98 missing data, mainly due to the COVID-19 pandemic).

### 3.2. Workplace-Based Clinical Assessment

The median overall score was 14.6 [13.2–15.9]/20 and did not differ between years (*p* = 0.06), including across each theme of evaluation (theme 1: 16.0 [14.3–16.7] *p* = 0.79; theme 2: 15.0 [13.0–16.0] *p* = 0.42; theme 3: 13.7 [12.6–15.4] *p* = 0.35). The median overall score differed across the internship sites (*p* = 0.02). Pairwise comparisons showed only a limited number of significant differences, with higher scores observed in cardiological follow-up care and rehabilitation, and lower scores in pediatrics compared with other clinical pharmacy services (Supplementary Table S1).

A significant difference in the overall score was observed between the groups according to the timing of the senior clinical pharmacy internship (*p* < 0.001). Scores were lower when the internship was completed in the first (S1) or second (S2) position compared with the fourth (S4) position (S1 vs. S4 *p* = 0.03; S2 vs. S4 *p* = 0.04). Regarding the assessment domains, no significant differences were observed for themes 1 and 3 (*p* = 0.30 and *p* = 0.06), whereas a significant difference was found for theme 2 (*p* = 0.01, Table 1, full details of the pairwise comparisons are available in Supplementary Materials Table S2).

Students with prior clinical experience performed better than those without (14.9 vs. 14.3, *p* < 0.001). This difference was not observed for theme 1 (*p* = 0.45) but was significant for themes 2 and 3 (*p* = 0.01 for both, Table 1).

Students in the hospital track performed better than those in the community pharmacy track (14.7 vs. 14.1, *p* < 0.001). This difference was not observed for theme 1 (*p* = 0.53) but was significant for themes 2 and 3 (*p* < 0.001 for both).

### 3.3. Case-Based Oral Assessment

The median score was (14.8 [13.0–16.0]) and did not differ between years (*p* = 0.67). Scores differed significantly according to the student’s training track (*p* < 0.001). Students in the hospital track achieved higher scores (15.2 [13.6–16.7]) than those in the community pharmacy track (14.9 [13.2–16.0], *p* = 0.01) and the industry track (13.6 [11.6–15.2], *p* < 0.001). Moreover, students in the community pharmacy track had better scores than those in the industry track (*p* < 0.001).

### 3.4. Student’s Satisfaction

**Figure 3:**
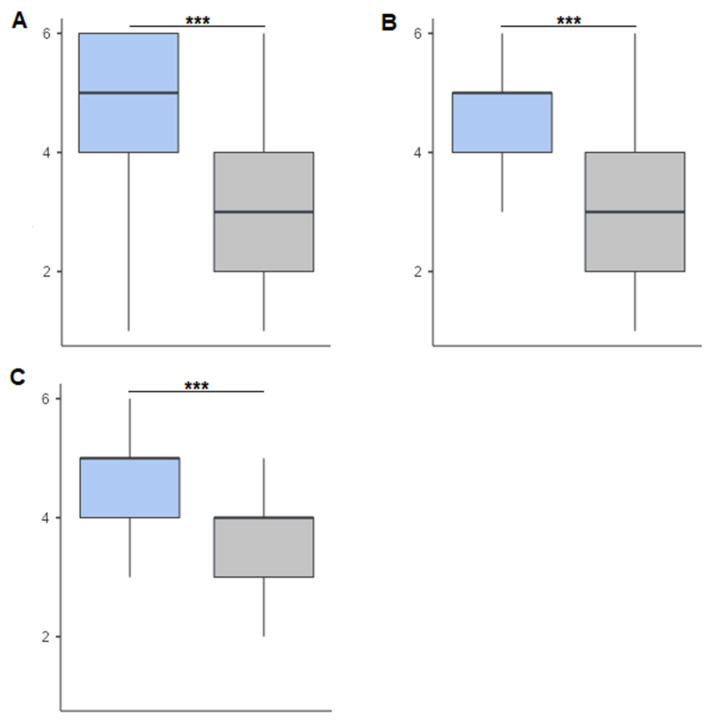
Students’ evaluations of clinical rotation. In blue is the evaluation of internship with direct pharmacist supervision. In grey, this is the evaluation of internship without direct pharmacist supervision. (**A**): Evaluation of the supervision quality (*n* = 442); (**B**): evaluation of the student’s impact (*n* = 528); (**C**): evaluation of acquired knowledge (*n* = 528); ***: *p* < 0.001.

Based on 528 evaluations, supervision quality was rated higher during internships with pharmaceutical supervision compared with those without (5 [4–6] vs. 3 [2–4], *p* < 0.001). Students also reported a greater perceived impact during internships with pharmaceutical supervision compared with those without (5 [4–5] vs. 3 [2–4], *p* < 0.001). Similarly, they reported better knowledge acquisition during internships with pharmaceutical supervision than during those without (5 [4–5] vs. 4 [3–4], *p* < 0.001) (Figure 3).

## 4. Discussion

Over a ten-year period, this study evaluated nearly one thousand pharmacy students through two complementary assessment modalities: the workplace-based clinical assessment, conducted in real-life clinical settings, and the case-based oral assessment. The overall median score for the workplace-based clinical assessments remained stable across years, though performance varied according to the timing of the internship, previous clinical experience, and the student’s chosen track. The case-based oral assessments showed comparable trends, with higher scores among students in the hospital track, reflecting the consistency of the competency-based approach across training modalities. Student satisfaction was high, especially during internships with direct pharmaceutical supervision, emphasizing the educational impact of structured mentorship and experiential progression. Altogether, these findings confirm the pedagogical relevance of integrating both real-life and case-based assessments as complementary tools to strengthen clinical reasoning and professional identity in pharmacy education.

The introduction of a workplace-based clinical assessment represents a major step forward in the professionalization of pharmacy education. From the beginning of their clinical placements, students are exposed to real patient situations requiring the integration of pharmacotherapeutic knowledge, communication skills, and clinical reasoning. In parallel, a large proportion of students are engaged in part-time work, most often in community pharmacy settings, which further increases their exposure to real-world practice and reinforces the articulation between academic training and professional experience. Several studies have shown that early clinical exposure, when supported by structured supervision, increases students’ confidence and autonomy, and relevance of their pharmaceutical analyses [10]. The present evaluation model aligns with this approach, positioning students as active contributors within the healthcare team rather than passive observers. This immersive participation enables a deeper understanding of the clinical pharmacist’s responsibilities and their impact on medication safety and quality of care [11].

Our longitudinal data indicate that overall scores improved as students progressed through their rotation sequence. Those who completed the clinical pharmacy internship later in the academic year obtained higher scores than those starting with it. This trend reflects a cumulative learning effect, confirming the benefit of a progressive and alternating structure between university courses and clinical practice to consolidate both theoretical and transversal competencies [12].

Mandatory rotation across hospital wards serves a dual purpose: ensuring exposure to diverse clinical settings and maintaining continuity of pharmaceutical activities without overwhelming supervisors. The quality of clinical supervision largely depends on supervisor availability and continuity of student–supervisor relationships [13]. Within our model, pre-defined rotations distribute the educational workload evenly and maintain consistent service coverage by progressively autonomous students. This system also fosters pedagogical alignment between wards, enhancing the consistency of teaching methods and assessment criteria. At the institutional level, it contributes to balancing pharmacists’ multiple missions in care, education, and research, in line with system-oriented models of health professional education aimed at strengthening healthcare organizations [14].

The development of a self-assessment questionnaire following a professional practice rotation is a cornerstone of reflective learning. It enables students to measure their progress and critically appraise their own performance. Workplace-based clinical assessment helps recognize the gap between perceived and demonstrated competence. Such an approach is consistent with contemporary educational frameworks in health professions education, which conceptualize self-assessment as a guided, reflective process embedded in experiential learning [15]. As an area for improvement, the implementation of a pre-test at the beginning of the academic year and a post-test at its completion could provide a more robust assessment of the evolution of students’ competencies and allow a more precise characterization of changes in self-efficacy over time.

The combination of self- and peer-assessment creates a valuable triangulation of perspectives. The simultaneous evaluation by a senior pharmacist and a pharmacy resident ensures balanced, formative feedback and increases reliability and validity [16]. Research from other health professions supports this dual approach as a mechanism to foster metacognition, self-regulation, and continuous improvement [17,18].

Our experience exemplifies a coherent implementation of competency-based education principles [19]. Combining a workplace-based clinical assessment with a case-based oral assessment ensures a holistic view of performance: the former captures competence in action, the latter evaluates analytical and communication skills. The integration of both dimensions into the student’s portfolio supports longitudinal competency development and aligns with standards for quality assurance in pharmacy education [20].

Students expressed a high level of satisfaction with internships supervised by clinical pharmacists, particularly regarding supervision quality, perceived utility, and learning outcomes. These results confirm the critical role of dedicated clinical supervision in promoting engagement and motivation [21]. Moreover, the observed performance differences across internship timing, prior experience, and track of specialization underscore the need for tailored pedagogical strategies. Several areas for improvement can be identified, such as strengthening supervisor training in formative assessment and feedback methods, extending evaluation to outcome-based indicators, such as the clinical relevance of students’ pharmaceutical interventions and the quality of interprofessional collaboration, and integrating structured reflection on the assessment of psychosocial competencies, which are currently being progressively implemented within the curriculum. Finally, dissemination of this model across French faculties could foster national harmonization of clinical evaluation practices, consistent with global frameworks for transformative health professional education [14]. While rooted in the French system, this model is highly adaptable to international settings. In resource-limited environments, the workplace-based clinical assessment could be focused on high-risk wards, while the case-based oral assessment can serve as a low-cost, high-impact tool for clinical reasoning development.

To successfully implement and adapt this formative assessment model, institutions should consider the following concrete actions. First, implement a mandatory “pre-briefing/debriefing” sequence for each workplace-based clinical assessment. During the de-briefing, supervisors should provide immediate, structured feedback using the “sandwich method” (highlighting a strength, identifying a specific area for improvement, and concluding with a corrective action plan) to foster reflective learning. Second, to reduce subjectivity and potential halo effects, institutions should provide short annual training sessions (e.g., 1 h webinars) for clinical pharmacists. These sessions should focus on the use of the 5-point competency grid and behavioral anchors to promote more objective scoring. Third, moving beyond performance scores, institutions could integrate the “clinical relevance of pharmaceutical interventions proposition” performed by students to their pharmacist or medical residents as a key metric. Tracking the number and acceptance rate of these pharmaceutical interventions performed by the student during the rotation would provide a direct measure of their impact on patient safety and quality of care. Finally, developing a longitudinal digital portfolio where students can record their self-assessments and supervisor feedback across all rotations would facilitate continuous monitoring of professional development and easier identification of students in difficulty.

Although its single-center design may limit external generalizability, this work presents a large, real-life evaluation spanning ten academic years and nearly one thousand students. The retrospective analysis relies on routinely collected educational data, exposing the results to missing data (e.g., during the COVID-19 period). However, the relatively low proportion of missing data (10.5%) and the consistency of the results across academic years suggests that these missing data are unlikely to have substantially altered the overall findings. The workplace-based clinical assessment is embedded in routine care, enhancing ecological validity and capturing authentic communication, reasoning, and decision-making skills. Assessments were conducted jointly by a senior clinical pharmacist and a pharmacy resident. While this approach likely improved internal consistency, the lack of quantitative inter-rater reliability measures remains a limitation and may affect the robustness of the assessment process. In addition, potential halo or context effects cannot be excluded. The parallel case-based oral assessment provides a complementary lens on analytical and communication competencies, enabling triangulation across modalities. Although we accounted for several factors, residual confounding from unmeasured variables (e.g., individual case complexity, variations in ward workload, or the specific pedagogical style of different supervisors) may have influenced student performance and should be considered when interpreting the results. Satisfaction measures are self-reported and susceptible to response and ceiling biases, as students may tend to over-report positive or negative experiences or cluster their ratings at the higher or lower end of the scale.

## 5. Conclusions

This study aimed to address the lack of standardized, competency-based evaluation methods in French hospital pharmacy training by assessing the performance, determinants, and acceptability of a combined workplace-based and case-based assessment model. Over ten academic years, this model demonstrated stable performance outcomes, with higher scores among students with prior clinical experience and those in hospital training tracks. Student satisfaction was consistently high, particularly in settings with direct pharmaceutical supervision, highlighting the importance of structured mentorship.

By integrating workplace-based, case-based, and self-assessment approaches, this approach provides a comprehensive evaluation of clinical competencies and supports reflective learning and professional development. These findings support the relevance and sustainability of this model for competency-based clinical pharmacy education and its potential for broader implementation.

## Figures and Tables

**Table 1:** Detailed results obtained regarding time of realization, previous clinical experience and track.

Parameters of the Workplace-Based Clinical Assessment Evaluated	Rotation in Which the Senior Clinical Internship Was Completed					Previous Clinical Experience During the Fifth Year			**Student’s Chosen Track**		
	1	2	3	4	*p*-Value	No	Yes	*p*-Value	**Ambulatory**	**Hospital**	*p*-Value
Total	14.2 [13.1–15.6]	14.3 [13.0–15.4]	14.8 [13.6–16.0]	15.0 [13.7–16.1]	0.004	14.3 [13.1–15.4]	14.9 [13.5–16.1]	<0.001	14.3 [13.1–15.5]	14.9 [13.6–16.1]	<0.001
Theme 1	15.4 [14.3–16.6]	16.0 [14.3–16.7]	15.4 [14.3–16.6]	16.0 [14.3–17.1]	0.298	15.4 [14.3–16.6]	16.0 [14.3–17.0]	0.565	15.4 [14.3–16.6]	16.0 [14.3–17.0]	0.512
Theme 2	15.0 [13.0–16.0]	15.0 [13.0–16.0]	15.5 [14.0–16.0]	16.0 [14.0–16.0]	0.010	15.0 [13.0–16.0]	15.5 [14.0–16.0]	0.007	15.0 [13.0–16.0]	15.8 [14.0–16.0]	<0.001
Theme 3	13.7 [12.3–15.4]	13.7 [12.0–14.9]	14.3 [12.6–15.4]	14.3 [12.6–15.7]	0.064	13.7 [12.0–14.9]	14.3 [12.6–15.4]	0.012	13.7 [12.0–15.4]	14.3 [12.6–15.9]	0.004

Data are expressed as the median [IQR], *n* = 598.

## Data Availability

The datasets generated during and/or analyzed during the current study are available from the corresponding author upon reasonable request.

## Supplementary Materials

The following supporting information can be downloaded at https://www.mdpi.com/article/10.3390/pharmacy14040090/s1. Table S1. Pairwise comparison of workplace-based clinical assessments according to the internship site. Table S2. Pairwise comparison of workplace-based clinical assessments according to the internship completion position.

## Abbreviations

The following abbreviations are used in this manuscript:

AM	Ante Meridiem
COVID-19	Coronavirus Disease 2019
PM	Post Meridiem
