# Supplementary material for: Performance, Determinants, and Acceptability of a Clinical Pharmacy Assessment in Hospital Pharmacy Education
Source: Pharmacy (Basel). 2026 Jun 24;14(4):90. doi: 10.3390/pharmacy14040090 (PMC13415117; doi:10.3390/pharmacy14040090)
Supplement: Supplementary file 1 [file pharmacy-14-00090-s001.zip › pharmacy-4278095-supplementary.pdf]

Supplementary data

Performance, Determinants, and Acceptability of a Clinical Pharmacy Assessment in Hospital Pharmacy Education

Sébastien Chanoine [1,2,3T*], Quentin Perrier [1,T], Elisa Vitale [1,2], Arnaud Tanty[1], Benoît Allenet[1,2] and Pierrick Bedouch[1,2]

[1] Univ. Grenoble Alpes, Pharmacy Department, Grenoble Alpes University Hospital, Grenoble, France
[2] Univ. Grenoble Alpes, CNRS, UMR 5525, VetAgro Sup, Grenoble INP, CHU Grenoble Alpes, TIMC, Grenoble, France
[3] Univ. Grenoble Alpes, INSERM U1209, CNRS UMR 5309, Institute for Advanced Biosciences (IAB), Team of Environmental Epidemiology Applied to Development and Respiratory Health, Grenoble, France
[T] Equally contribution
* Correspondence: sebastien.chanoine@univ-grenoble-alpes.fr

Table S1. Pairwise comparison of workplace-based clinical assessments according to the internship site

Dwass-Steel-Critchlow-Flinger pairwise comparison

Sites		W	p
Diabetology	Infectiology	-2.767	0.680
Diabetology	Nephrology	-0.651	1.000
Diabetology	Cardiological rehabilitation	7.275	< .001
Diabetology	Geriatrics	4.091	0.125
Diabetology	Hepato-gastroenterology	-1.022	1.000
Diabetology	Hematology	1.240	0.999
Diabetology	Pneumology	-1.882	0.964
Diabetology	Rheumatology	1.128	0.999
Diabetology	Hematology day hospital	1.070	1.000
Diabetology	Pediatrics	-5.633	0.003
Infectiology	Nephrology	2.569	0.771
Infectiology	Cardiological rehabilitation	7.678	< .001
Infectiology	Geriatrics	5.275	0.009
Infectiology	Hepato-gastroenterology	1.030	1.000
Infectiology	Hematology	3.361	0.382
Infectiology	Pneumology	0.688	1.000
Infectiology	Rheumatology	3.167	0.477
Infectiology	Hematology day hospital	2.681	0.721
Infectiology	Pediatrics	-4.092	0.125
Nephrology	Cardiological rehabilitation	7.450	< .001
Nephrology	Geriatrics	4.269	0.090
Nephrology	Hepato-gastroenterology	-0.859	1.000
Nephrology	Hematology	1.656	0.986
Nephrology	Pneumology	-1.708	0.982
Nephrology	Rheumatology	1.296	0.998
Nephrology	Hematology day hospital	1.377	0.997
Nephrology	Pediatrics	-6.150	< .001
Cardiological rehabilitation	Geriatrics	-2.996	0.564

Dwass-Steel-Critchlow-Flinger pairwise comparison

Sites		W	p
Cardiological rehabilitation	Hepato-gastroenterology	-6.622	< .001
Cardiological rehabilitation	Hematology	-3.960	0.158
Cardiological rehabilitation	Pneumology	-7.544	< .001
Cardiological rehabilitation	Rheumatology	-5.044	0.016
Cardiological rehabilitation	Hematology day hospital	-4.100	0.123
Cardiological rehabilitation	Pediatrics	-6.946	< .001
Geriatrics	Hepato-gastroenterology	-3.991	0.150
Geriatrics	Hematology	-1.384	0.997
Geriatrics	Pneumology	-5.041	0.016
Geriatrics	Rheumatology	-2.213	0.897
Geriatrics	Hematology day hospital	-1.582	0.990
Geriatrics	Pediatrics	-5.945	0.001
Hepato-gastroenterology	Hematology	2.237	0.890
Hepato-gastroenterology	Pneumology	-0.680	1.000
Hepato-gastroenterology	Rheumatology	1.852	0.968
Hepato-gastroenterology	Hematology day hospital	1.813	0.972
Hepato-gastroenterology	Pediatrics	-4.338	0.078
Hematology	Pneumology	-2.811	0.658
Hematology	Rheumatology	-0.551	1.000
Hematology	Hematology day hospital	-0.117	1.000
Hematology	Pediatrics	-5.842	0.002
Pneumology	Rheumatology	2.524	0.790
Pneumology	Hematology day hospital	2.207	0.899
Pneumology	Pediatrics	-4.380	0.072
Rheumatology	Hematology day hospital	0.309	1.000
Rheumatology	Pediatrics	-5.332	0.008
Hematology day hospital	Pediatrics	-5.148	0.012

Table S2. Pairwise comparison of workplace-based clinical assessments according to the internship completion position

**Dwass-Steel-Critchlow-Flinger pairwise comparison**				
	**Rotation**		**W**	**p**
Overall	1	2	-0.00466	1.000
	1	3	3.45948	0.069
	1	4	3.88566	0.031
	2	3	3.36156	0.082
	2	4	3.76364	0.039
	3	4	0.47077	0.987
Theme 1	1	2	0.740	0.954
	1	3	0.293	0.997
	1	4	2.438	0.311
	2	3	-0.449	0.989
	2	4	1.721	0.616
	3	4	2.178	0.414
Theme 2	1	2	0.716	0.958
	1	3	3.686	0.045
	1	4	3.590	0.054
	2	3	3.041	0.138
	2	4	2.959	0.156
	3	4	-0.147	1.000
Theme 3	1	2	-0.896	0.921
	1	3	1.960	0.508
	1	4	2.376	0.334
	2	3	2.855	0.181
	2	4	3.210	0.105
	3	4	0.498	0.985
